# Sequential biases on subjective judgments: Evidence from face attractiveness and ringtone agreeableness judgment

**DOI:** 10.1371/journal.pone.0198723

**Published:** 2018-06-11

**Authors:** Jianrui Huang, Xianyou He, Xiaojin Ma, Yian Ren, Tingting Zhao, Xin Zeng, Han Li, Yiheng Chen

**Affiliations:** Guangdong Key Laboratory of Mental Health and Cognitive Science, Center for Studies of Psychological Application, School of Psychology, South China Normal University, Guangzhou, China; Universita degli Studi di Udine, ITALY

## Abstract

When people make decisions about sequentially presented items in psychophysical experiments, their decisions are always biased by their preceding decisions and the preceding items, either by assimilation (shift towards the decision or item) or contrast (shift away from the decision or item). Such sequential biases also occur in naturalistic and real-world judgments such as facial attractiveness judgments. In this article, we aimed to cast light on the causes of these sequential biases. We first found significant assimilative and contrastive effects in a visual face attractiveness judgment task and an auditory ringtone agreeableness judgment task, indicating that sequential effects are not limited to the visual modality. We then found that the provision of trial-by-trial feedback of the preceding stimulus value eliminated the contrastive effect, but only weakened the assimilative effect. When participants orally reported their judgments rather than indicated them via a keyboard button press, we found a significant diminished assimilative effect, suggesting that motor response repetition strengthened the assimilation bias. Finally, we found that when visual and auditory stimuli were alternated, there was no longer a contrastive effect from the immediately previous trial, but there was an assimilative effect both from the previous trial (cross-modal) and the 2-back trial (same stimulus modality). These findings suggested that the contrastive effect results from perceptual processing, while the assimilative effect results from anchoring of the previous judgment and is strengthened by response repetition and numerical priming.

## Introduction

When people make judgments of single physical attributes of stimuli presented in a series, sequential effects often occur [1,2] in which judgments of the current stimulus are influenced by the preceeding items. The most widely explored sequential effects are assimilative effects and contrastive effects. A contrastive effect refers to a shift in the response in the direction opposite of the value of the previous item, and an assimilative effect refers to a shift in the response in the direction towards the previous response.

Biases in perceptual judgments resulting from preceeding trials have been extensively studied by psychophysicists [1–8]. For example, Holland and Lockhead (1968) conducted a study using absolute judgments of loudness and found that the stimulus on the immediately preceding trial had an assimilative effect on the current response, whereas the preceding stimuli on two to five trials back all had a contrastive effect on the current response. In a series of four experiments, Ward found both assimilative and contrastive sequential effects in category judgment, absolute identification, magnitude estimation, and cross-modality matching experiments [9]. In a study of taste judgments, both preceding responses and preceding stimuli were shown to affect current judgments of sweetness intensity [10]. Specifically, assimilation was found for responses: higher rating of sweetness on the previous trial led to a higher rating on the current trials. A contrastive effect was found for stimulus sweetness objectively defined: higher sweetness on the previous trial led to lower ratings of sweetness on the current trial.

To better understand these sequential effects found in psychophysical experiments, Jesteadt, Luce, and Green (1977) put forward the following regression model:

$$J_{n}=\gamma +\alpha_{0}P_{n}+\alpha_{1}P_{n-1}+\beta_{1}J_{n-1}+\varepsilon ,$$ (1)

where P_n_ is the value of the stimulus presented on the current trial, P_n−1_ is the value of the stimulus on the previous trial, J_n−1_ is the rating made on the previous trial, γ is a constant related to the average response magnitude used by the participant, and ε is the usual Gaussian error term. Both previous response and stimulus can exert either a contrastive effect or assimilative effect on the judgment on current stimulus.

Sequential effects are also found in more complex, real-world judgments. Matthews and Stewart (2009) found that price judgments also follow the regression model (Eq 1) established in psychophysical research[11]. When participants were asked to estimate the price of chairs presented in sequence, their judgment on the current trial was biased towards the value of their previous response (assimilation) but away from the actual price of the previous item (contrast). Although there is some multicollinearity in the regression model because the J_n−1_ and P_n−1_ predictors are correlated, the largest variance inflation factor (VIF) of a total of 81 regressions is 3.03, which is far less than 10, indicating acceptable multicollinearity [12,13].

Pegors et al.(2015) used a novel sequential rating design to measure the effects of the previous stimulus and the previous response on face attractiveness judgments. They alternated the type of judgment (darkness of hair vs attractiveness) on every other trial to obtain estimates of the bias attributable to the attractiveness of the previous face as well as the bias attributable to the orthogonal response. They found both an assimilative effect resulting from the rating of the darkness of the hair and a contrastive effect resulting from the attractiveness value.

What mechanism can account for contrastive effects in subjective sequential decision-making tasks like attractiveness judgments? Pegors et al. (2015) suggested an underlying visual perceptual mechanism based on the finding that the contrastive stimulus bias was strengthened by increasing the duration of the previous stimulus, following from earlier research that demonstrated that perceptual aftereffects for simple visual attributes processed early in the cortical hierarchy increase logarithmically with adapting duration and decay exponentially with test duration [14]. However, evidence from other studies showed that when participants made judgments of multi-dimensional stimuli, some dimensions produce contrast and some produce assimilation [15], which is contrary to a simple perceptual mechanism. Additionally, Pegors et al. (2015) failed to observe sequential effects when participants rated the temperature of places and face attractiveness alternately on a 1–8 Likert-type scale, and they argued that the absence of effects was a result of the change of stimulus category (two separate semantic/perceptual categories) rather than other factors, which was contradictory to some early studies. Conversely, another study showed that the attractiveness rating of a given face or object assimilated toward the rating of the preceding trial and held that the sequential effect can be extended to the domain of subjective decision-making[16]. Furthermore, researcher found that in mixed-modality psychophysical scaling (e.g., light-sound-light-sound), participants’ responses were assimilated to the immediately previous response (different modality stimulus) but contrasted with the stimulus (same modality) two trials back in the sequence [17,18]. However, whether assimilation occurs in the domain of mixed-modality subjective decision-making (e.g., face attractiveness-ringtone agreeableness) in real world needs further confirmation.

There were several possible interpretations of the observed assimilative effect. Some researchers suggested that when the participants were unsure of their judgment, then they simply repeated the previous response [19]. Thus, the tendency to repeat was the key point to assimilation. Some argued that there was genuine assimilation rather than mere repetition in perceptual identification study [20]. Others believed that the previous item was used as a point of reference for the current judgment and people anchored and adjusted [21,22]. Some researchers even argued that meaningless numbers may cause priming effect on the next trial [23–26]. In terms of subjective judgment, Matthews and Stewart (2009) found that price judgment was assimilated toward the preceding judgment but providing feedback of the true price largely decreased the assimilation, which suggested anchoring to the most recent item led to assimilation. Taubert et al. (2016) designed a binary task in which participants typically make binary decisions (attractive or unattractive) about each face in a sequence of unfamiliar faces. They found that participants were more likely to rate a face as attractive when the preceding face was attractive than when it was unattractive. Later they conducted an experiment with stimuli alternating randomly between upright and inverted orientation which disrupted almost all perceptual processes underlying face perception and found that the assimilative effect diminished when the two consecutive faces were incongruent in orientation. Therefore, they attributed the assimilative effect in attractiveness judgment to visual perception. In contrast, Pegors et al. (2015) demonstrated that assimilative response bias was not strengthened by increased display duration of previous stimulus, which suggested that assimilative effect was not served by perceptual mechanism. In a word, the underlying mechanism of assimilative effect was still in debate and remained unclear.

In this article, we conducted five experiments to explore the mechanisms of contrastive and assimilative effects in the sequential subjective judgments. We first examine whether there are both contrastive effects and assimilative effects in judgments of face attractiveness (Experiment 1) and ringtone agreeableness (Experiment 2) to test the universality of sequential biases in the domain of subjective judgments. Based on the previous literature, we hypothesized that both assimilative effects and contrastive effects would occur simultaneously. Next we examined the influence of the provision of feedback (Experiment 3), the participant’s response modes (Experiment 4), and cross-modal stimuli (Experiment 5) on sequential effects, expecting to shed light on the mechanisms of sequential effects. Besides, previous studies have found that provision of feedback after participants enter their judgments masked the perception of the previous stimulus [27] as well as the numerical priming on the previous rating [11]. If the sequential effects diminished or eliminated significantly when feedback is provided, then sequential effects at least partly result from the perception of the previous stimulus or the anchoring of the previous judgment. In terms of response collecting methods, compared to keyboard response, oral response reduces the tendency of action repetition for the relatively flexibility in mouth muscle movement, which allows us to examine whether assimilative effect can be accounted for by action repetition tendency. Additionally, the cross-modal stimuli allow us to provide a more unified view on whether the sequential effects are modality-dependent due to their simple perceptual mechanism.

## Experiment 1

Face attractiveness, which is of great importance in interpersonal interaction, is a holistic visual trait that we often use to make first-pass assessments of people as we associate this feature with romantic viability, sociability, and health (see reviews [28]). Sequential biases on face attractiveness were prevalent [29–36], but they were little understood. Experiment 1 aimed to primarily find out whether face attractiveness ratings made in sequence were influenced by the true attractiveness values of the previous faces and the ratings given to the previous face. We used the standard sequential judging paradigm in Experiment 1, in which participants judged the attractiveness of a series of faces one by one on a scale of 1 (least attractive) to 8 (most attractive).

### Method

#### Participants

Twenty-eight female undergraduate students at South China Normal University, age ranging from 18 to 26 (*M*age = 20.77, *SD* = 1.62), participated in the experiment. All of them were right-handed with normal vision or vision corrected to normal. The data from three participants were excluded due to ceiling or floor effects in the baseline rating of the faces, defined as a mean rating more than 2 SD above (1 participant) or below (2 participants) the mean rating of all the faces across all the participants, leaving a total of 25 participants included in the data analyses. The study was approved by the Academic Committee of the School of Psychology at South China Normal University. All participants gave written informed consent before participating in the study.

#### Stimuli

Facial stimuli were obtained from Glasgow Unfamiliar Face Database, with a wide range of attractiveness. From the 169 available images of Caucasian males aged 18–30, 98 stimuli with 80% or more approval rate of the five researchers were primarily selected. Faces with unusual expressions, accessories, or uncommon hairstyles were further excluded, leaving 88 pictures in the final stimulus set. All the 88 stimuli were of Caucasian males, forward facing, with neutral expressions and normal hairstyles. The control of race, gender and age was to minimize the influence of irrelevant variables [32,35,37], and using young male faces also was expected to promote their female counterparts’ aesthetic desire (Burleson, Hall, & Gutierres, 2015). A large body of evidence has demonstrated that people are better at remembering own-race faces in comparison with faces of another race (e.g., [38]), and attractiveness ratings are remarkably consistent across cultures[39–41]. Thus, the choice of Caucasian instead of Asian faces was to avoid the possible interference of experienced-based familiarity and recognition. The pictures only included the body parts above chin, resized to place on about 400*400 pixel blank backgrounds.

#### Procedure

In the pre-evaluation, the attractiveness of facial stimuli was first rated by 30 participants not participating in the formal experiment. Participants rated images on a 1–8 Likert-type scale, with 1 referring to the least attractive; 8 referring to the most attractive. To diminish sequential bias in pre-evaluation, the stimuli were presented in random order, and ratings were made under no time pressure. Each picture was rated twice in two blocks by the same participant to obtain test-retest reliability. The data from four participants were excluded due to low reliability, ceiling or floor effects. The average rating across the remaining 26 raters for each item served as the baseline stimulus value in the formal experiment.

**Fig 1:**
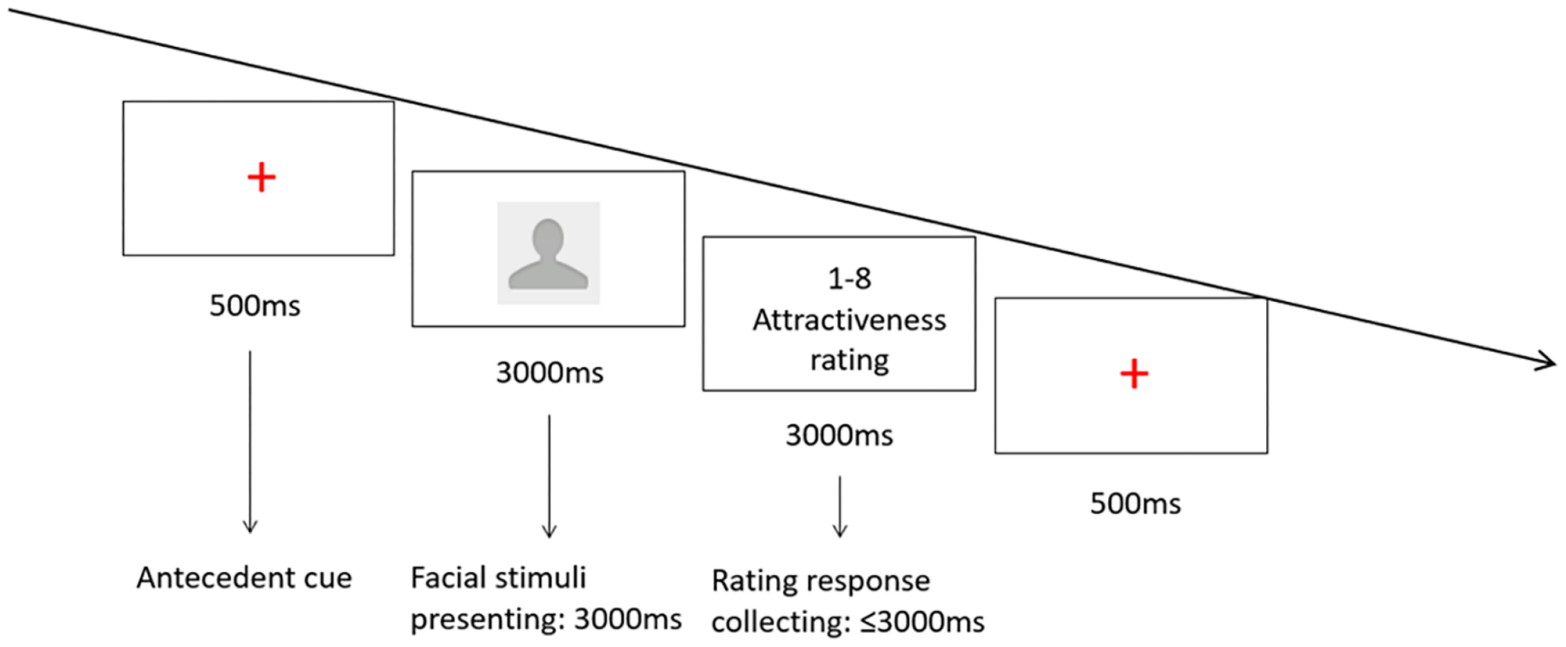
Design for Experiment 1. A sequential design for face attractiveness judgment. Participants rated the attractiveness of each male face on a 1–8 Likert-type scale. Note that we use real facial stimulus to replace the blank profile picture in the experiment.

The experiment was programmed with E-prime 1.0. In the formal experiment, 30 participants were tested in quiet testing cubicles, viewing the screen from a distance of approximate 55 cm. They were required to rate the facial stimuli according to their attractiveness on the same 1–8 Likert-type scale as in pre-evaluation. Ratings used the number keypad on the right of the keyboard instead of the separated number keys across the keyboard to reduce finger travel time. After participants read the instructions and made sure they understood them, they first performed a practice session of 8 trials (the photos used here were not for the formal experiment) before the formal experiment. Each trial began with a fixation cross in the middle of the screen for 500ms. Then a facial stimulus was presented for 3000ms, followed by a response-collecting window for the participants to enter their ratings. As soon as a response was entered, or 3000ms elapsed without any response detected, the fixation cross was presented and the next trial began (see Fig 1). The experiment consisted of two blocks, which shared the same set of 80 stimuli. Stimuli were presented in random order within each block.

### Results and discussion

Attractiveness ratings in the pre-evaluation were averaged across raters for each stimulus to determine its attractiveness value, the sequential bias of which was supposed to have been filtered out in this way, making it a relatively unbiased value. The mean stimulus value of all face stimuli across all 26 raters was 3.36 (*SD* = 0.69). Furthermore, we calculated the correlation coefficient of response time and the face attractiveness rating for all 26 raters but found no significant correlation between response time and the score of face attractiveness rating, which implied that response time was not an interference variable. Therefore, all the stimuli including visual stimuli and audio stimuli were presented for the same 3000ms in the following experiments. Participants entered their ratings after the stimuli disappeared within another 3000ms time window. Of the 25 participants, 20 responded within the window on all trials, whereas 5 participants missed between 1–3 of the 160 trials.

In the main experiment, we tested the 1-back sequential effects by using the following polynomial regression equation (Eq 1):

$$R_{t}=\beta_{0}+\beta_{1}S_{t}+\beta_{2}R_{t-1}+\beta_{3}S_{t-1}+\varepsilon ,$$ (2)

where R_t_ represents participant’s response to the current trial, S_t_ represents the stimulus value of the current trial, R_t-1_ and S_t-1_ represent the attractiveness rating and stimulus value for the previous trial separately, and ε is the error term.

**Fig 2:**
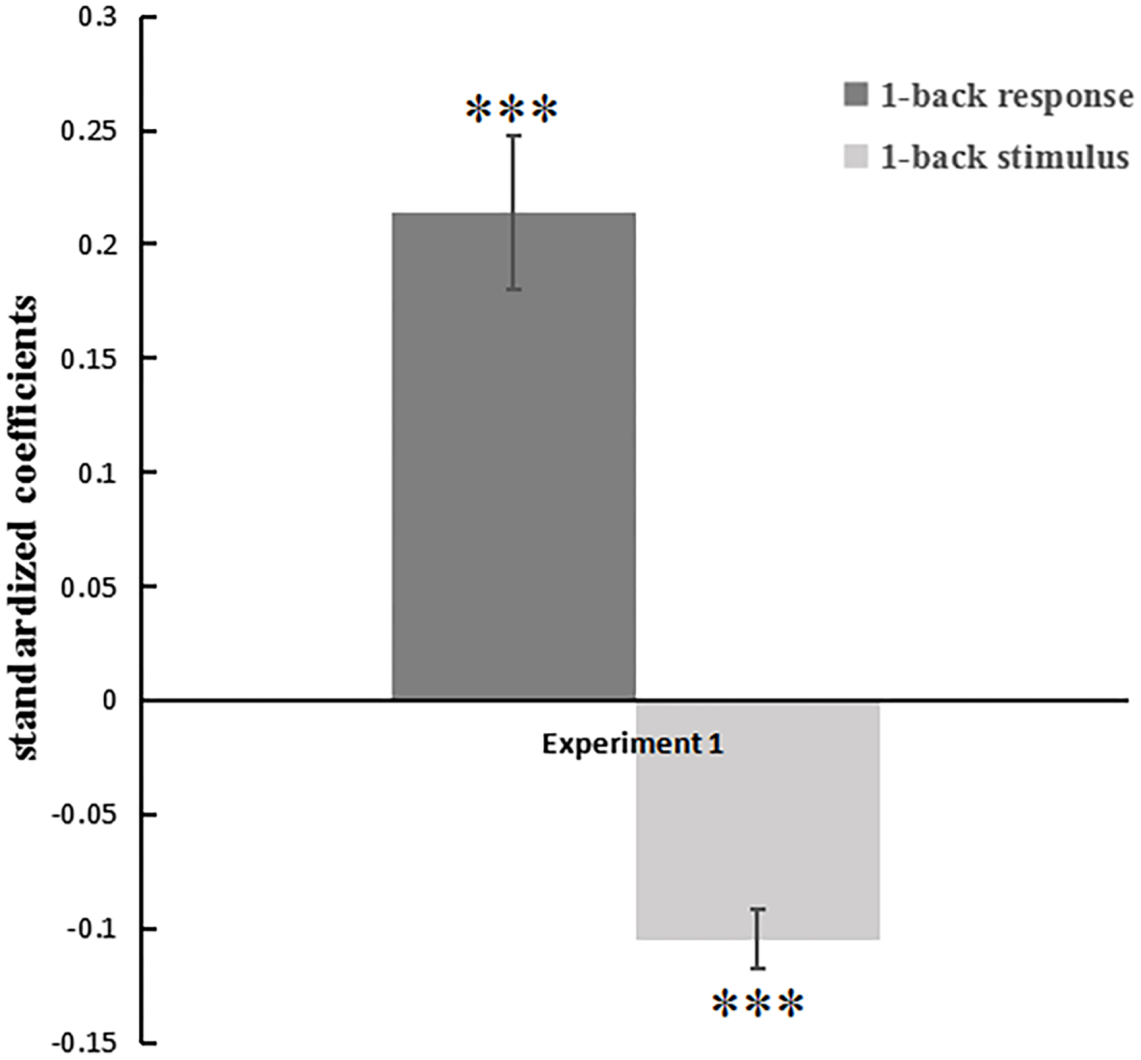
Face attractiveness ratings were regressed against the previous response and the previous stimulus (the stimulus value). In Experiment 1, both elements of the previous trials significantly predicted the current judgments. The previous response positively predicted the current judgments (an assimilative effect), while the previous stimulus negatively predicted the current judgments (a contrastive effect). Error bars represent the standard error of the mean. *p<0.05, **p<0.01, ***p<0.001.

Beta estimates of the previous stimulus and response predictors were extracted for each subject-specific regression model, which were presented in Table 1. Twenty-four of the 25 coefficients of R_t-1_ were positive (14 of them were significant), indicating assimilation to the previous response. All participants (6 of the coefficients of S_t-1_ were significant) showed a contrastive effect of the attractiveness value of the previous stimulus. Then as Lorch and Myers suggested, we conducted one sample t-test to examine whether the mean of the regression coefficients collected from the 25 participants reliably differed from zero [42]. Results from testing these betas against zero revealed that there was a significant contrastive effect of the previous stimulus (β3 = -.11, *t*[24] = -8.23, *p* < .001; see Fig 2). That is, faces were judged as more attractive if they were preceded by faces with lower attractiveness value, and vice versa.

**Table 1:** Regression coefficients for Experiment 1.

participant	Unstandardized				Standardized			
	int	Rt-1	St-1	St	Rt-1	St-1	St	Adjusted R^2^
1	0.636**	0.447***	-0.157	1.211***	0.447	-0.066	0.493	0.434
2	-3.264*	0.066	-0.400	2.212***	0.066	-0.127	0.677	0.420
3	0.331	0.093	-0.036	0.550***	0.091	-0.023	0.355	0.118
4	-0.523	0.079	-0.100	0.948***	0.080	-0.058	0.548	0.284
5	0.165	0.431***	-0.108	0.844***	0.437	-0.067	0.519	0.463
6	-0.968	0.199*	-0.362*	1.245***	0.201	-0.186	0.639	0.367
7	-1.260*	0.098	-0.200	0.913***	0.100	-0.011	0.486	0.240
8	0.640	0.155	-0.032	0.786***	0.155	-0.023	0.552	0.324
9	0.633	0.168*	-0.336	1.011***	0.169	-0.121	0.364	0.147
10	0.128	-0.035	-0.179	0.585***	-0.035	-0.140	0.459	0.188
11	-0.525	0.064	-0.362	1.560***	0.064	-0.133	0.558	0.290
12	-1.026*	0.176*	-0.316*	1.103***	0.177	-0.191	0.668	0.432
13	-0.568	0.259***	-0.089	1.062***	0.254	-0.045	0.531	0.343
14	-0.133	0.177*	-0.262**	0.816***	0.177	-0.211	0.655	0.448
15	-1.106	0.335***	-0.356	1.531***	0.335	-0.123	0.534	0.362
16	-0.371	0.120	-0.035	1.164***	0.121	-0.018	0.592	0.367
17	-0.891	0.291**	-0.455*	1.288***	0.289	-0.189	0.539	0.327
18	-0.491	0.704***	-0.077	0.546***	0.714	-0.029	0.204	0.527
19	0.689	0.103	-0.319*	1.138***	0.101	-0.173	0.623	0.374
20	-0.250	0.308***	-0.192	1.081***	0.309	-0.057	0.340	0.199
21	0.700	0.252**	-0.221	0.684***	0.253	-0.140	0.435	0.231
22	-0.761	0.468***	-0.407**	1.051***	0.474	-0.190	0.491	0.380
23	-1.327	0.023	-0.255	1.553***	0.024	-0.099	0.599	0.341
24	-1.634**	0.211**	-0.269	1.282***	0.216	-0.130	0.615	0.428
25	-0.671	0.125	-0.133	1.185***	0.125	-0.068	0.609	0.378
*M*	-0.474	0.213***	-0.226***	1.094***	0.214***	-0.105***	0.523***	0.337
*SD*	0.907	0.167	0.129	0.372	0.168	0.064	0.115	0.103

*p<0.05,

**p<0.01,

***p<0.001

We also observed a significant and positive result toward an assimilative influence from the previous response (β2 = .21, *t*[24] = 6.35, *p* < .001). That is, faces were judged as more attractive if they were preceded by faces which were also judged as more attractive, and vice versa. The averaged beta (standardized) across all participants was -0.11 (all of 25 betas were negative) for the contrastive effect and 0.21 (24 of 25 betas were positive) for the assimilative effect (see Table 1). Our findings are in line with earlier studies that assimilative effect and contrastive effect existed simultaneously [10,11,34,43].

**Fig 3:**
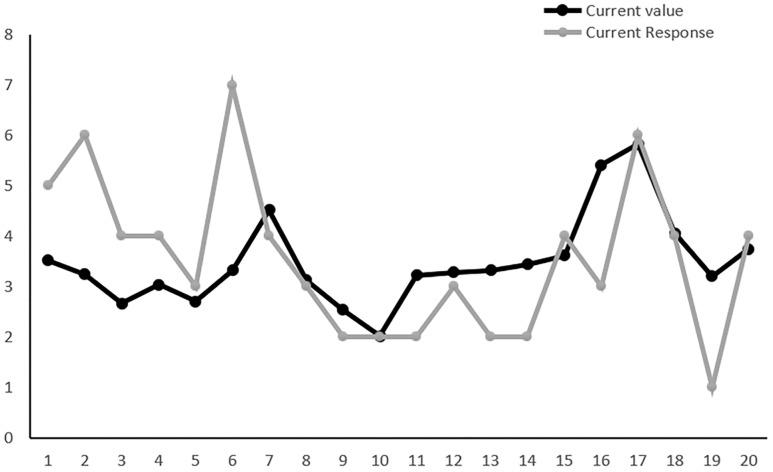
The data were extracted from one of the participants on the first 20 trials in Experiment 1, which showed the response pattern of one participant.

To better understand the assimilative effect and contrastive effects, we present the data from one of the participants on the first 20 trials in Experiment 1 (see Fig 3). As we can see from the line chart, there was an obvious rise in stimulus value between Trial 5 and 6, but the rise in rating did not parallel the rise in attractiveness. The face on Trial 6 received unmatched high rating due to its huge contrast in attractiveness to the previous face. Similarly, when the face on Trial 19 showed a decrease in attractiveness compared with Trial 18, the participant’s rating dropped more drastically. These are typical examples of contrastive effects. In contrast, as the stimulus value on Trial 10 decreased from Trial 9, participant’s response remained at the same level, which can be seen as an example of an assimilative effect.

One concern is that there is some multicollinearity in the regression model because the predictors R_t-1_ and S_t-1_ are correlated. Multicollinearity increases the standard errors of the regression coefficients (although the coefficients remain unbiased estimators), reducing the likelihood that a particular coefficient will be significant. Therefore, we formally tested for multicollinearity by examining the variance inflation factor (VIF) of each of the independent variables. In general, a VIF larger than 10 indicates multicollinearity between independent variables, whereas a VIF less than 10.0 indicates acceptable multicollinearity (e.g., [12,13]). Of all 75 (each regression had three independent variables) VIFs in Experiment 1, the highest was 1.77, well below the critical value. We examined VIFs in all our experiments; all were less 10 and thus we conclude that multicollinearity between R_t-1_ and S_t-1_ was not a problem for the analysis.

Another concern is the appropriateness of the calculation method of face attractiveness value. Some may argue that it is inappropriate to use average ratings to define the attractiveness of previously presented faces. There are objective computational measures of symmetry, for instance, that correlate highly with attractiveness. However, we assume that other factors such as averageness also contribute largely to attractiveness of a certain face, which make it less possible to set the objective standard for attractiveness of a face. Importantly, participant’s rating of the attractiveness of a certain face in the formal experiment correlated highly with the attractiveness value we obtained in the pre-evaluation (r = .44, p < .001). Even if there are precise computational models of facial attractiveness judgments, our interest lies in the sequential biases rather than face attractiveness per se. Moreover, some may argue that it is inappropriate to define the face attractiveness using the average ratings of a different sample of participants rather than those who participated in the formal experiment. We reran the analyses, and the result showed the same pattern no matter we calculate the attractiveness value based on different samples of participants or the same. When the attractiveness values are obtained from the participants in the formal experiment, estimated betas for R_t-1_ and S_t-1_ were 0.20 (*SE* = .03; *t* = 7.55, *p*< .001) and -0.15 (*SE* = .03; *t* = -5.03, *p*< .001) respectively, suggesting significant assimilative and contrastive effects.

## Experiment 2

When it comes to musical stimuli, sequential bias was also found [44]. That is, musical stimuli are evaluated more positively if they follow bad stimuli than otherwise, vice versa [45]. Whether trial-by-trial sequential biases found in the face attractiveness judgments also occurs in auditory aesthetic can be an interesting issue to be examined. Experiment 2 aimed to characterize sequential biases in auditory stimuli judgments. The same design as Experiment 1 was used, with the exception that auditory stimuli (ringtones) were used instead of visual stimuli (faces).

### Method

#### Participants

Twenty-eight female participants were recruited from the same population as in the Experiment 1. None of the participants had previously participated in Experiment 1. The mean age of the 28 participants was 21.06 (SD = 1.16), and they were all right handed. All the participants had normal hearing ability in both ears.

#### Stimuli

Forty-four ringtones from the iPhone and Android ringtone libraries were downloaded from the Internet. We selected ringtones randomly with the goal of achieving a representative sampling. Ringtones were edited with Cool Edit pro V2.1. They were first converted into WAV format and then a 3000-ms span audio segment was randomly selected and cut out from each ringtone as the stimulus. All audio stimuli were adjusted to a 44.1-khz sampling rate and 16-bit bit rate, and were played in stereo effect at a consistent volume through headphones. Four of the 44 stimuli were used in the practice session and the remaining 40 were used in the formal experiment. A 500-ms visual cue (picture of a trumpet) was presented before every upcoming ringtone stimulus. This stimulus played the same role as the fixation cross in previous visual-stimulus experiments.

#### Procedure

**Fig 4:**
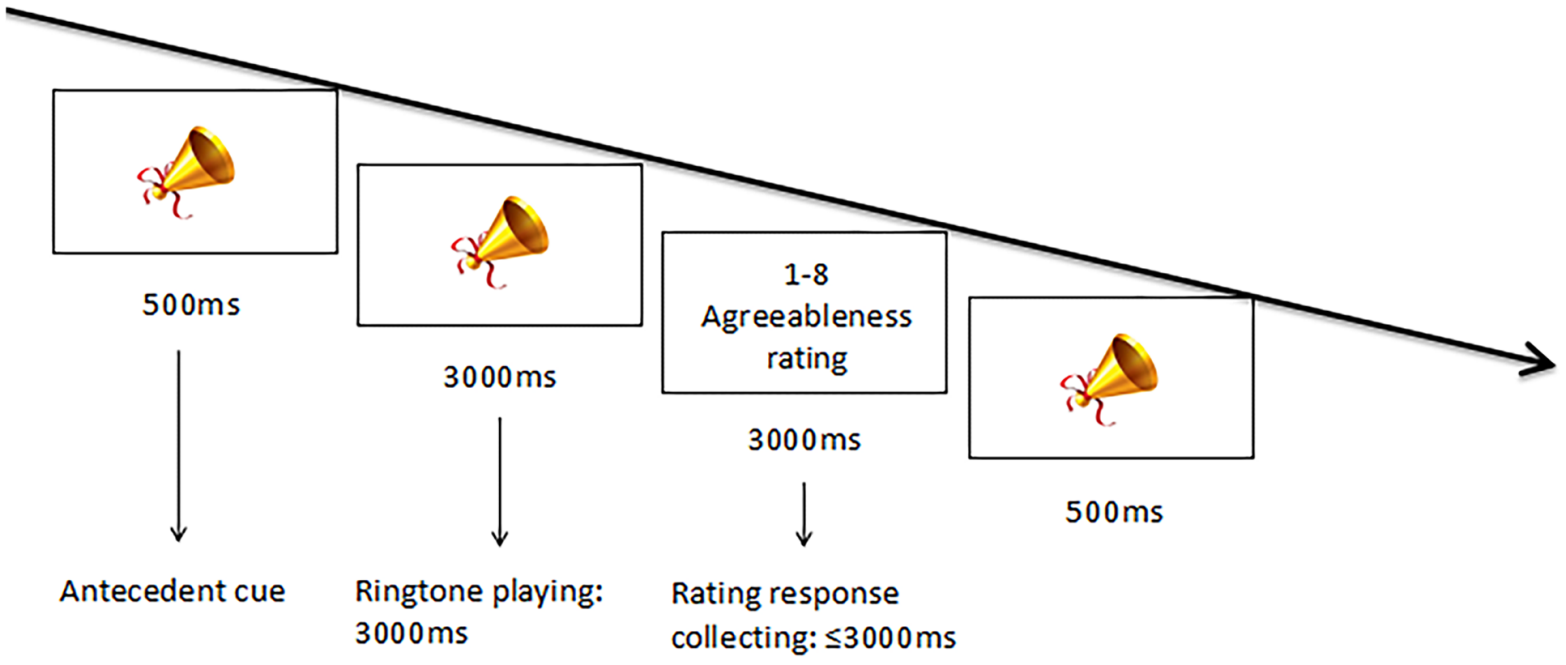
Design for Experiment 2. The design was matched to that of Experiment 1, except that the stimuli were auditory ringtones instead of visual faces, and the antecedent cue was replaced by a picture of a trumpet. Participants rated the agreeableness of each stimulus on a 1–8 Likert-type scale.

The procedure was parallel to the previous visual-stimulus experiment, except that the fixation cross was replaced by a picture of a trumpet and the facial stimuli were replaced by ringtone audio stimuli. After reading the instructions, participants performed a practice session of 4 trials before the formal experiment. Each trial began with a 500-ms visual cue. Then an auditory ringtone stimulus was played for 3000ms, followed by a response-collecting screen for the participants to enter their agreeableness ratings (see Fig 4). The experiment consisted of two blocks, each with 40 trials of different auditory stimuli played in random order.

Since the auditory stimuli were all presented for 3000ms in a random order across participants, the average rating was used as the baseline measure of stimulus value in data analysis.

### Results and discussion

The agreeableness rating for each ringtone was averaged across raters to calculate its baseline stimulus value. The average stimulus value of all ringtones across all 28 raters was 4.21 (*SD* = 1.11).

**Fig 5:**
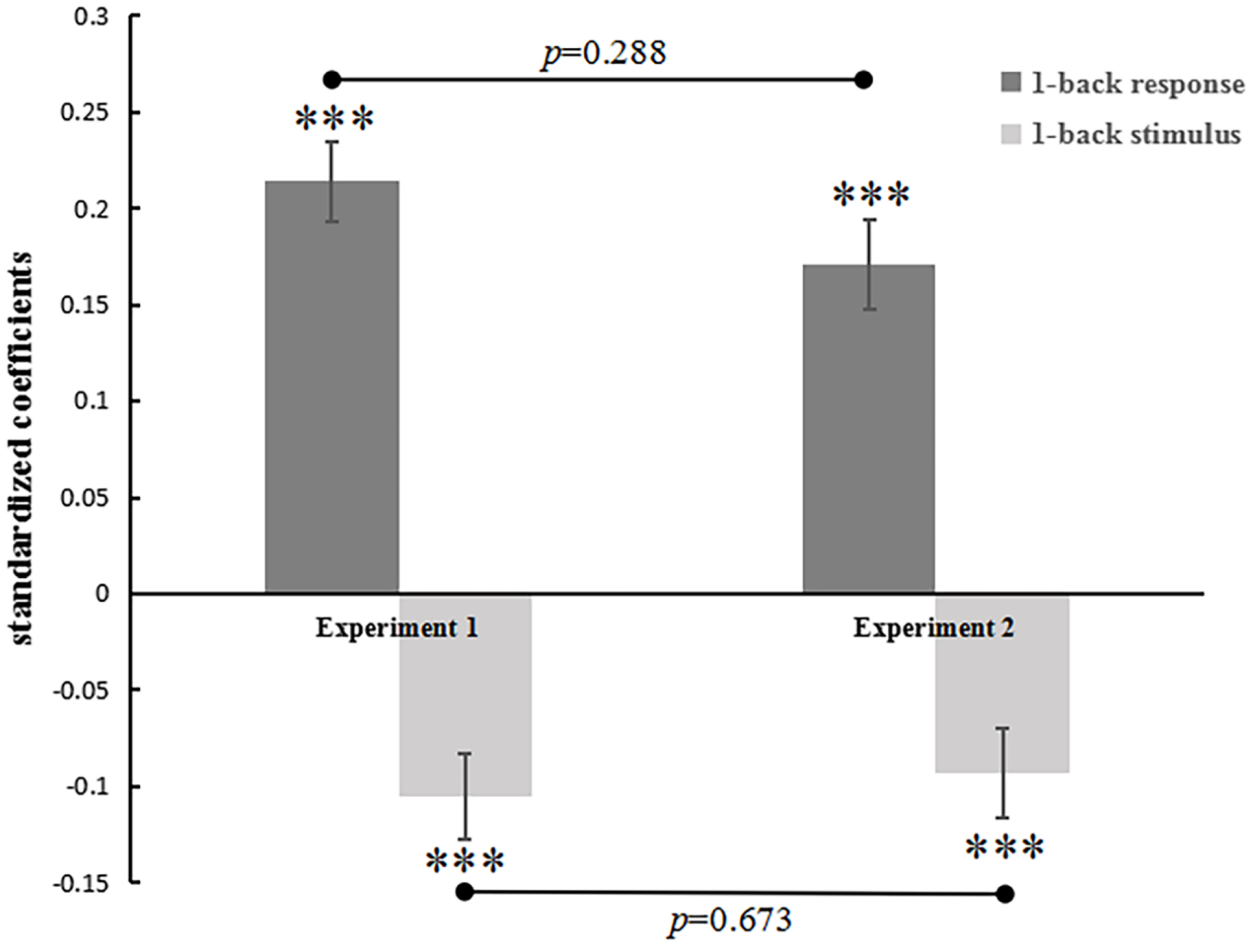
Ringtone agreeableness ratings were regressed against the previous response and the mean agreeableness rating of the previous stimulus (baseline stimulus value). Both elements of the previous trial significantly predicted the judgment on the current trial. The previous response positively predicted the current judgments (an assimilative effect), while the previous stimulus agreeableness negatively predicted the current judgment (a contrastive effect). There was no significant difference of the size of both assimilative effect and contractive effect between Experiment 1 and Experiment 2. Error bars represent the standard error of the mean. *p<0.05, **p<0.01, ***p<0.001.

We tested the 1-back sequential effects by using the same regression model as in previous experiment (Eq 2). Beta estimates of the previous stimulus and response predictors were also extracted for each subject-specific regression model. Then we conducted one sample t-test to examine each regression coefficient across all participants. Results from testing these betas against zero revealed that there was a significant contrastive effect with the previous stimulus (β3 = -.09, *t*[27] = -4.07, *p* < .001; see Fig 5). That is, ringtones were judged as more agreeable if they were preceded by ringtones with lower stimulus value, and vice versa. We also observed a significant and positive result towards an assimilative influence from the previous response (β2 = .17, *t*[27] = 7.40, *p* < .001). That is, ringtones were judged as more pleasing if they were preceded by ringtones which were also judged as more pleasing, and vice versa.

We conducted independent sample t-test on the beta estimates of the previous stimulus and response between Experiment 1 and Experiment 2. There was no significant difference between the two sets of data both in previous response (*t*[51] = 1.07, *p* = .288) and in previous stimulus (*t*[51] = -0.43, *p* = .673). However, null hypothesis significance test never enables us to accept the null hypothesis (H_0_). Therefore, we conducted Bayesian independent samples t-test (Cauchy prior, width 1.0) again on the two sets of data. Results showed that Bayes factor BF_01_ for R_t-1_ and S_t-1_ were 2.25 and 3.36 respectively (When the Bayes factor BF_01_ equals 20, the data are 20 times more likely under H_0_ than under H_1_.). According to convention, Bayes factors BF_01_ ranging from 1 to 3 provide anecdotal, from 3 to 10, moderate, and above 10, strong evidence in favor of the null hypothesis (H_0_). To summarize, there was no differences between facial attractiveness judgment and ringtone agreeableness judgment for both assimilative and contractive effects, implying that the mechanisms underlying these sequential effects were not affected by the change between visual and auditory modality.

In conclusion, the results of the above two experiments establish that both the assimilative effect and the contrastive effect are robust and consistent across both visual and auditory modalities. In experiment 3 we examine how whether sequential effects can be modulated by feedback, and in experiment 5 we examine whether sequential effects can be extended across-sensory modalities.

## Experiment 3

As mentioned above, some researchers assumed that the contrastive effect resulting from the previous stimulus might be caused by perceptual aftereffect. Then the provision of trial-by-trial feedback (i.e., the attractiveness value), which can mask the perception of the previous stimulus, [27] can test the assumption. In addition, since the trial-by-trial feedback interferes participants’ tracing of the previous rating [11], it also indicates whether the assimilative effect is influenced by the reduced chance of numerical priming. Experiment 3 aimed to test the influence of feedback on sequential attractiveness rating bias in a within-subject design. Experiment 3 was identical to Experiment 1 except that participants were told the average rating of each face after they entered their judgments.

### Method

#### Participants

Thirty-two female participants were recruited from the same population as in the previous experiments. None of the participants had previously participated in the study. The mean age of the 28 participants was 20.93 (SD = 1.02), and they were all right handed. All of them were right-handed with normal vision or vision corrected to normal.

#### Stimuli

The same stimuli were used as in Experiment 1.

#### Procedure

The procedure was identical to Experiment 1, except that the average stimulus value of each face obtained from the pre-evaluation was shown on the screen for 800 ms after the participants entered their ratings.

### Results and discussion

**Fig 6:**
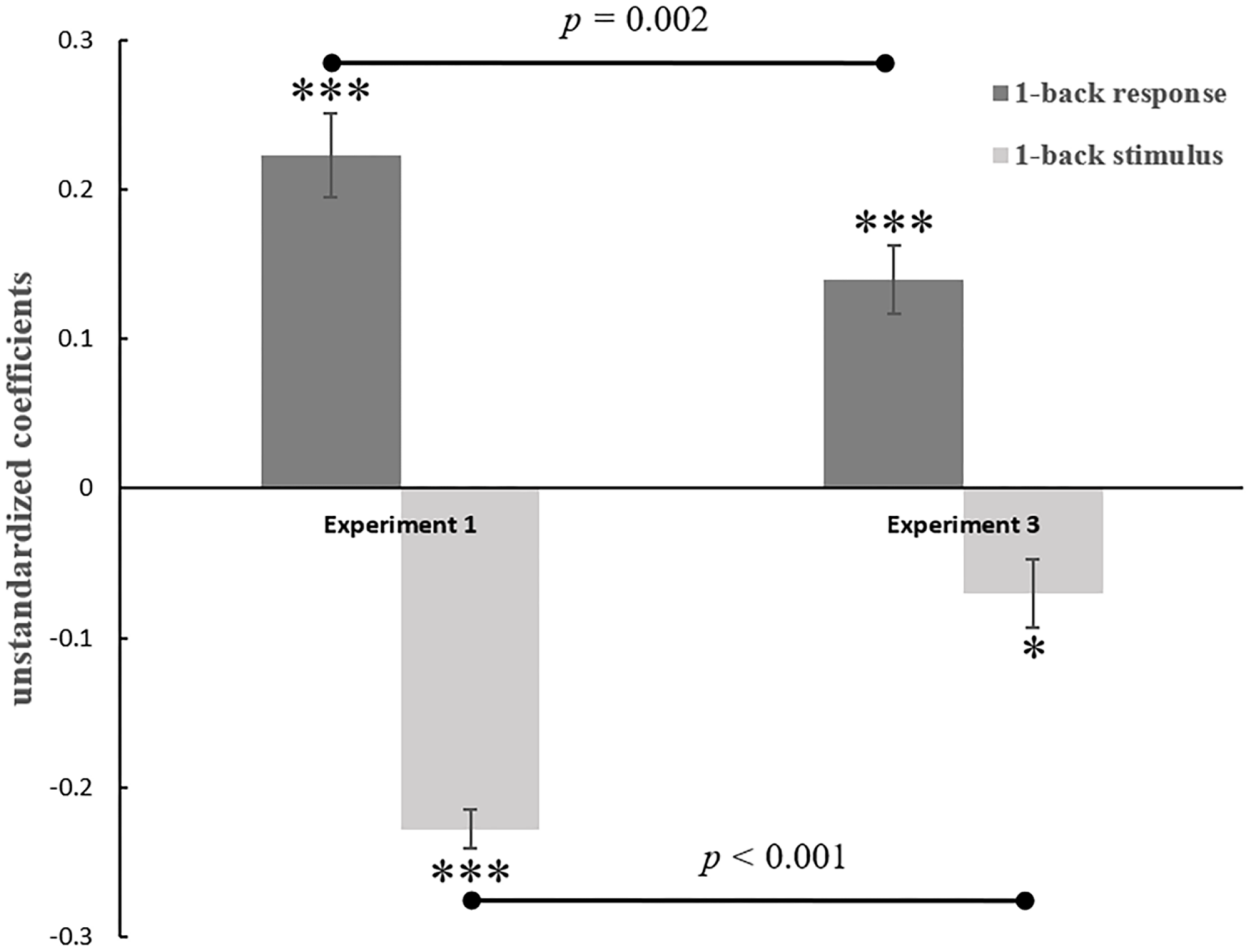
In Experiment 3, both previous response and previous judgment significantly predicted the current judgment. The size of both the assimilative effect and contrastive effect in Experiment 3 were significantly smaller than in Experiment 1. Error bars represent the standard error of the mean. *p<0.05, **p<0.01, ***p<0.001.

We tested the 1-back sequential effects by using the same regression model as in previous experiment (Eq 2). Beta estimates of the previous stimulus and response predictors were also extracted for each subject-specific regression model. We found that under the feedback provision condition, both the assimilative effect (β2 = .14, *t*[31] = 7.33, *p* < .001; see Fig 6) and the contrastive effect (β3 = -.07, *t*[31] = -2.07, *p* = .047; see Fig 6) were significant. Furthermore, we conducted a paired-sample t-test on the beta estimates of the previous stimulus and response between Experiment 1 and Experiment 3 to examine the impact of feedback on the sequential effects. According to the results, the trial-by-trial feedback did weaken both assimilative effect (*t*[55] = 3.28, *p* = .002, Cohen’s d = 0.88) and contrastive effect (*t*[55] = 6.26, *p* < .001, Cohen’s d = 1.67).

An earlier study showed that feedback largely eliminated the assimilation to the previous judgment and shifted the effects of the previous stimulus from weak contrast to weak assimilation [11]. In Matthews and Stewart’s study, participants estimated the prices of the shoes in a series. When no feedback was provided, there was a significant negative dependence on the previous item’s price but a significant positive dependence on the previous judgment. However, trial-by-trial feedback indicating each item’s true price reduced the effect of the previous judgment and shifted the effect of the previous item’s true price from contrast to assimilation. In our study, the provision of feedback eliminated the contrastive effect but we did not observe a shift towards weak assimilation across subjects. When individual participants were examined, we found that 8 of 25 participants showed significant assimilative effect of previous judgment and only 1 of 25 participants showed significant contrastive effect of the previous stimulus, with 9 of the participants showed weak and not significant assimilation to the previous stimulus (the feedback). Either way, these results were consistent with the idea that participants used the previous judgment as a point of reference in the domain of subjective decision-making (e.g., [30]).

As for the contrastive effect, one of the typical interpretations is that visual aftereffect, the result of our visual system constantly adapting to incoming stimulus information, results in the contrastive effect [34,46,47]. It seems that this interpretation explains our results well. Feedback destroys the contrastive effect owing to its destruction of the perceptual processing of the previous faces. In other words, providing feedback after participant enters his judgment on the face attractiveness eliminates the visual aftereffect on the next face. An alternative explanation for contractive effect is that a type of cognitive remapping takes place between faces and the scale itself. As Pegors et at. (2015) reviewed, “participants were remapping facial features to the ratings scale on a trial-by-trial basis, finely adjusting what rating they would give to what type of face on the basis of the recent attractiveness history.” However, this never happened because the feedback didn’t undermine the cognitive remapping anyway but it did destroy the contractive effect.

As for the assimilative effect, our findings are parallel to some psychophysical studies that participants use the previous item as a point of reference for the current judgment (e.g., [1,3,5]). Some researchers even argued that uninformative numerical anchors influenced a judgment even when people were not asked to compare this number to the target value [48–50]. In our study, the provision of the feedback masked the attractiveness rating of the previous stimulus, resulting in the diminishment of assimilative effect, which can be explained by the numerical priming to some extent. However, the assimilative effect was still significant when the numerical priming was destroyed, which may be the result of the tendency to repeat the previous judgment. Some researchers had demonstrated that participant just repeated his judgment when he was unsure of the current stimulus (e.g., [19,51]), which would be examined in the following experiments. In traditional response-collecting mode with a keyboard or mouse, a tendency of action repetition is much more likely to occur. However, participants can respond more freely when they just respond in voice [52–54]. We tried to further make clear of the nature of assimilative effect in the following experiments.

## Experiment 4

Experiment 4 examined whether the assimilative effect is influenced by action repetition. Oral responding rather than key-pressing was applied to avoid participants’ reluctance to change their rating across trials with relatively wide range in traditional key-pressing rating or mouse-clicking rating, which can relatively avoid the tendency of action repetition.

### Method

#### Participants

Twenty-eight female participants were recruited from the same population as in Experiment 1. The mean age of the 28 participants was 20.56 (SD = 1.26), and they were all right handed. None of them had participated in the previous studies.

#### Stimuli

The same 80 Caucasian male facial stimuli pictures were used as in Experiment 1.

#### Procedure

The procedure was identical to Experiment 1, except that while the response-collecting screen was present, participants were required to orally report their attractiveness rating to each facial stimulus. Their speech was recorded by a voice recorder, in order to avoid participants’ possible concerns and pressure from their subjective aesthetic standard being judged by any present experimenter. After giving oral responses, participants could press the space bar to start the next trial, which would also automatically start if 3000 ms elapsed without any space bar pressing. The speech-to-text transcription was later manually done by the experimenters to ensure correct recording.

### Results and discussion

**Fig 7:**
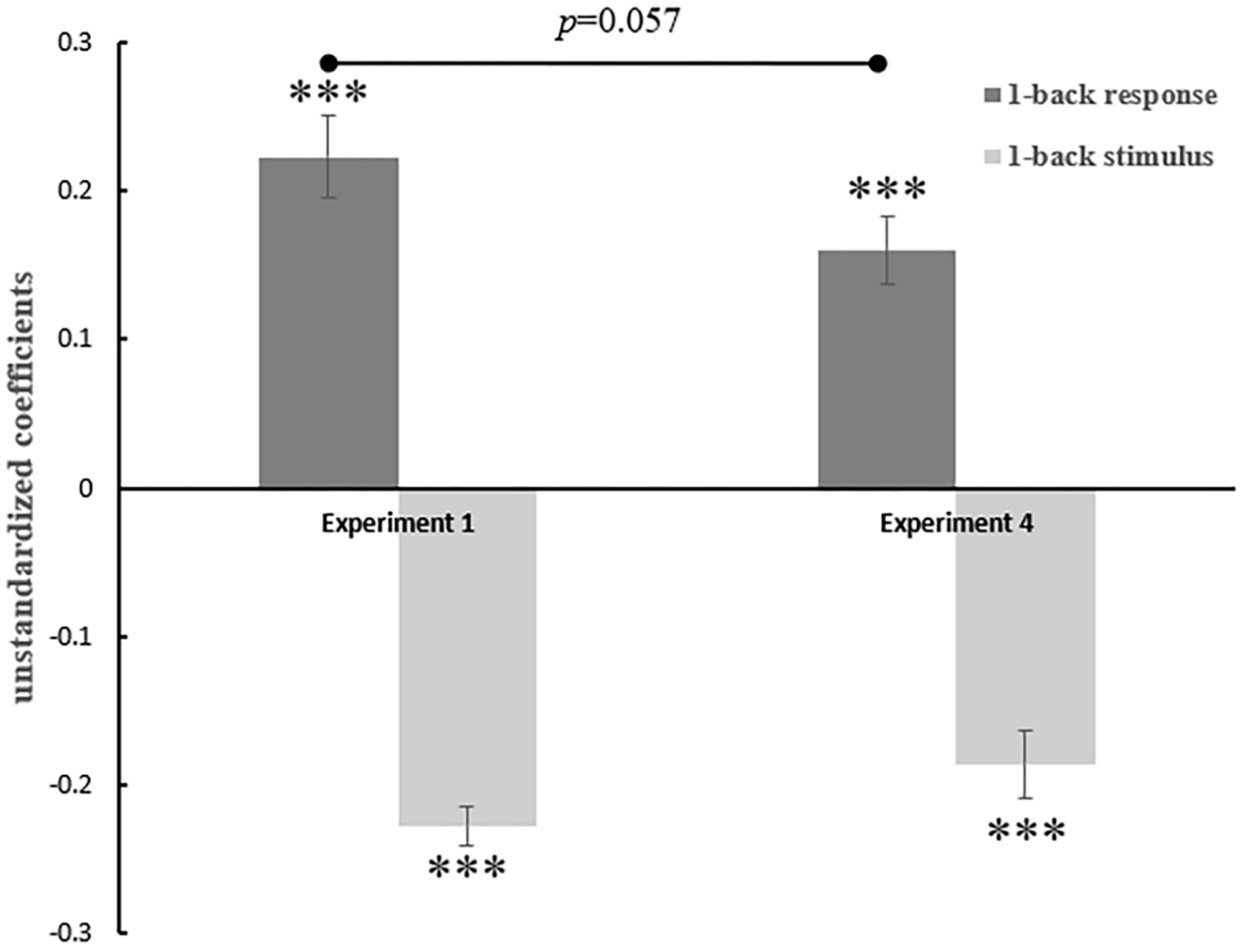
In the oral-response condition, both elements of the previous trials significantly predicted the current judgments. The previous response positively predicted the current judgments (an assimilative effect), while the previous stimulus negatively predicted the current judgments (a contrastive effect). The size of assimilative effect was significantly stronger when participants responded with a keyboard rather than responded orally. Error bars represent the standard error of the mean. *p<0.05, **p<0.01, ***p<0.001.

The same data analysis method as in Experiment 1 was used for Experiment 4 to examine the sequential effects. We found significant biases in face attractiveness ratings resulted from both the previous rating (β2 = .16, *t*[24] = 6.33, *p* < .001; see Fig 7) and the previous stimulus value (β3 = -.19, *t*[24] = -5.47, *p* < .001). Furthermore, we conducted independent-sample t-test on the beta estimates of the previous stimulus and response between Experiment 1 and Experiment 4. There was a marginal significant difference between the two sets of data in previous response (*t*[51] = 1.93, *p* = .057, Cohen’s d = 0.53), which illustrated that the assimilative effect in oral-responding condition was weaker compared with that of key-pressing condition. The results were in line with our hypothesis that participants’ tendency for action repetition can partially account for assimilation.

Although there are some concerns that whether the information in the verbal reports reflects thinking accurately and whether participants would change and alter the course of thought with verbal protocols (see reviews [54]). We believe that it’s appropriate to ask participants to respond orally in our experiments. In the history of facial attractiveness judgment, researchers asked participants to make their judgments either with keyboard [34,55] or mouse [56]. For example, participants were asked to rate the attractiveness of each face by clicking a mouse on a 7-point scale [30]. Taubert et al. (2016) asked participants to make dichotomous decisions about whether a face was attractive or not based on a brief glimpse of a profile picture and found the assimilative effect of the previous face. However, participants might just repeat their action or they are reluctant to move in a wide range. When participants made their judgments by orally report, they were able to respond without any restraint, and they could correct their answers when they thought their given answers were inappropriate, leading to weaker assimilative effect.

To summarize, Experiment 4 demonstrated that action repetition may contribute to part of the assimilative effect. In the subsequent experiment, we tested whether sequential effects would occur in cross-modality condition.

## Experiment 5

Experiment 5 used an alternative sequential presentation of both visual and auditory stimuli, where the same type of stimulus was presented on every other trial alternatively, to further determine whether sequential rating bias is identifiable under cross-modal situations. If the assimilative effect of the previous response is significant on cross-modal 1-back trials, then it can be explained by the priming of the previous ratings. If the assimilative effect is significant on 2-back trials that are of the same kind of stimulus modality, then it results from the anchoring of the same kind of stimulus. As for contrastive effect, if the value of the stimulus 1-trial back (of different modality) has no effect on the current rating, but the value of the stimulus 2-trial back (of the same modality) does, then we can draw the conclusion that the contrastive effect results from mere visual/auditory perception processing rather than high-level cognitive processing. In this experiment, the oral-responding rating method was applied to avoid the influence of action repetition tendency.

### Method

#### Participants

Thirty female participants who met the same requirements as in Experiment 2 were recruited. The mean age of the 30 participants was 21.32 (SD = 1.59), and they were all right handed. None of them had participated in the previous studies.

#### Stimuli

Both the visual and auditory stimuli used in previous experiments were adopted. Forty pictures were randomly selected from the total 80 facial stimuli from Experiment 1, along with all 40 auditory stimuli from Experiment 2.

#### Procedure

**Fig 8:**
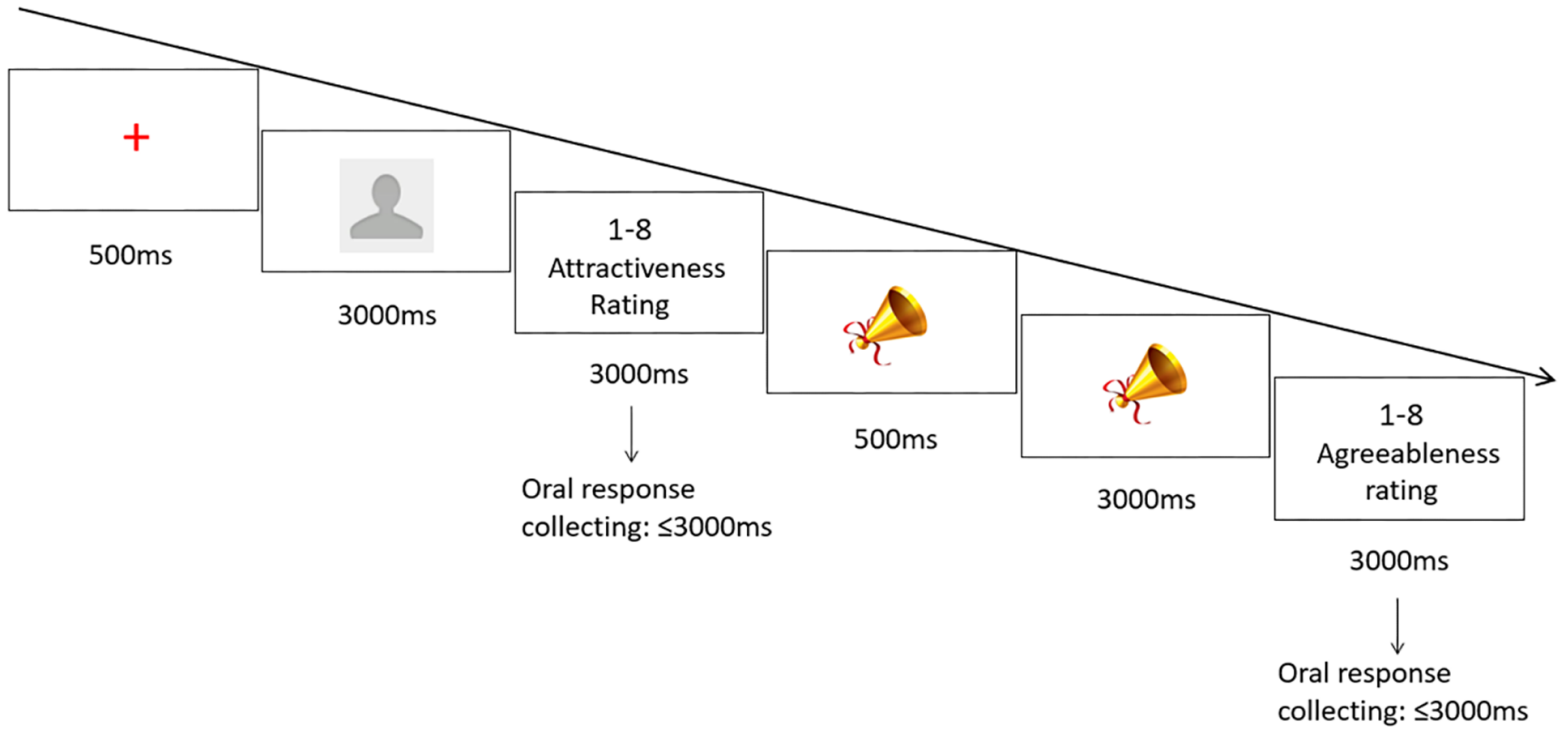
Alternating design in which participants rated either the facial attractiveness or musical agreeableness on a 1–8 Likert-type scale. Note that we use real facial stimulus to replace the blank profile picture in the experiment.

In the alternating sequential rating paradigm, visual and auditory stimuli alternated within each block, with a visual stimulus. presented first in each block. A fixation cross preceeded each visual stimulus; while a signal picture preceeded each auditory stimulus. Participants were required to orally rate the facial attractiveness or musical agreeableness (see Fig 8). The experiment had two blocks, with 80 trials of alternating stimuli (40 visual, 40 auditory) presented in random order in each block.

### Results and discussion

Visual and auditory stimuli alternated across trials, with the result that the stimulus presented on the 2-back trial was of the same type with stimulus on the current trial. We examined whether the judgments made on the current trial were influenced by both the 1-back and the 2-back trials with the following polynomial regression:

$${\text{R}}_{\text{t}}=\beta_{\text{0}}+\beta_{\text{1}}{\text{R}}_{\text{t}-2}+\beta_{2}{\text{S}}_{\text{t}-2}+\beta_{3}{\text{R}}_{\text{t}-1}+\beta_{4}{\text{S}}_{\text{t}-1}+\beta_{5}{\text{S}}_{\text{t}}+\varepsilon ,$$ (3)

Where *R*_*t*_ and *S*_*t*_ represent the response and stimulus value separately for the current trial, *R*_*t-2*_ and *S*_*t-2*_ represent the same values for the same type of stimulus two trials back, and *R*_*t-1*_ and *S*_*t-1*_ represent the response and stimulus value separately for the previous 1-back trial.

**Fig 9:**
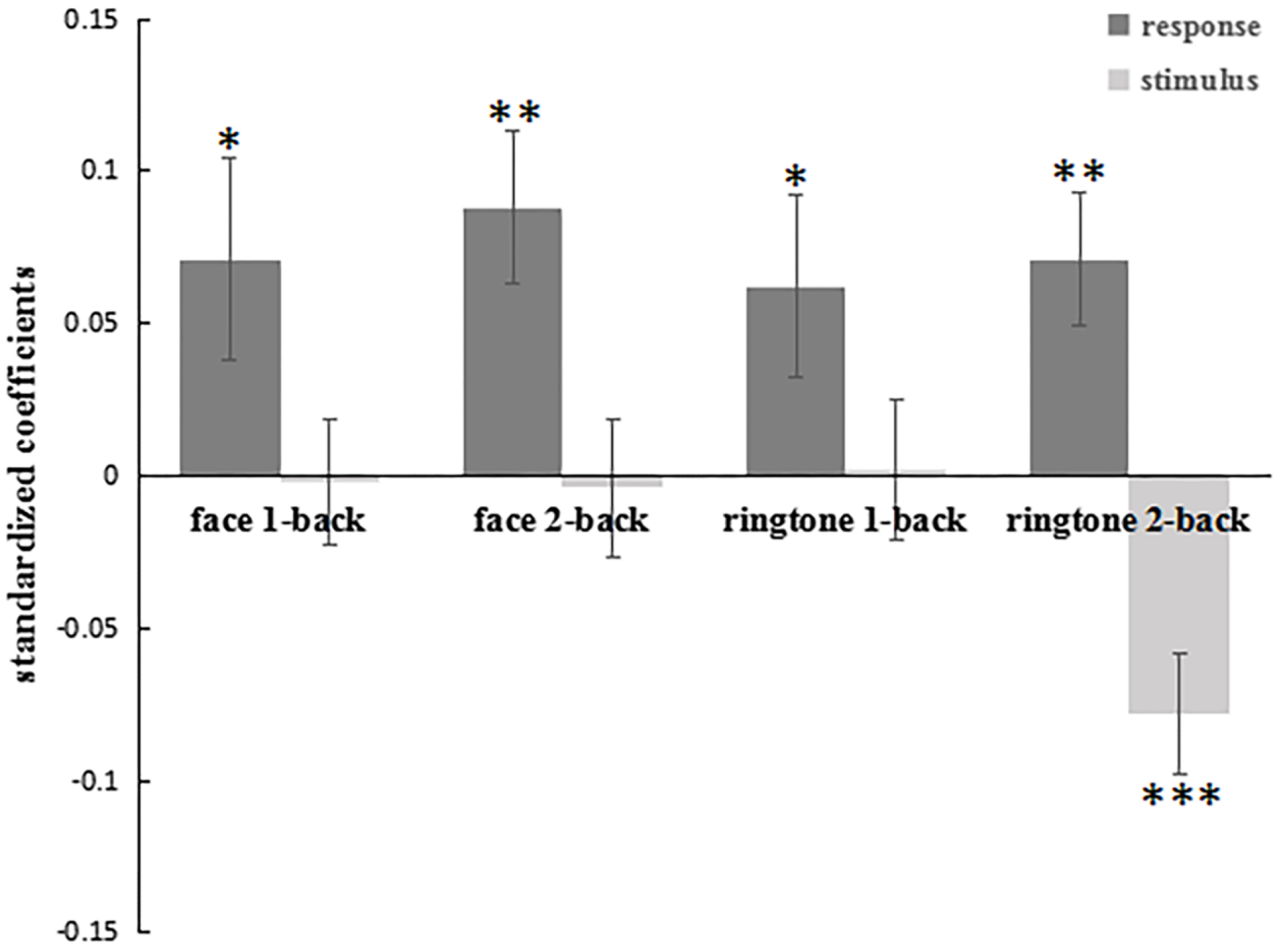
Ringtones’ agreeableness ratings and facial attractiveness ratings were regressed against the ratings and values of the previous 2 trials. The previous responses of both 1-back trials and 2-back trials positively predicted the current ratings (an assimilative effect). But only the agreeableness ratings of 2-back ringtones predicted (negatively) the current ratings. Error bars represent the standard error of the mean. *p<0.05, **p<0.01, ***p<0.001.

First, we examined the face attractiveness ratings. We found no significant contrastive effect of the previous stimulus (β4 = -.002, *t*[29] = -.10, *p* = .920; see Fig 9) but a marginally significant contrastive effect of the 2-back stimulus (β2 = -.04, *t*[29] = -1.17, *p* = .098). We also observed significant assimilative effects from both the 1-back response (β3 = .07, *t*[29] = 2.16, *p* = .039) and 2-back response (β1 = .09, *t*[29] = 3.58, *p* = .001).

Secondly, we examined the ringtone agreeableness ratings. For the 1-back trial, there was significant assimilative effect from the previous response (β3 = .06, *t*[29] = 2.10, *p* = .045; see Fig 9) but not a contrastive effect from the previous stimulus (β4 = .002, *t*[29] = .10, *p* = .918). For the 2-back trials, the rating of the ringtone positively predicted the rating on the current ringtone (β1 = .07, *t*[29] = 3.18, *p* = .003), while the value of the previous ringtone negatively predicted the rating of the current ringtone (β2 = -.08, *t*[29] = -3.94, *p* < .001).

For the insignificant results above, we computed Bayesian one sample t-tests to quantify the evidence in favor of the null hypothesis (H_0_). When the current trial was face, BF_01_ for S_t-1_ and S_t-2_ against 0 were 5.12 and 1.41 respectively. When the current trial was ringtone, BF_01_ for S_t-1_ against 0 were 5.18. According to convention, Bayes factors BF_01_ ranging from 1 to 3 provide anecdotal, from 3 to 10, moderate, and above 10, strong evidence in favor of the H_0_. Therefore, it was appropriate to accept the null hypothesis that S_t-1_ had no effect on the current trial. And face of 2-back trial had little impact on the current face.

Our results were consistent with some earlier studies (e.g., [16,19]. Since the ratings of the previous ringtone agreeableness positively predicted the judgments on the current face, the mechanisms of assimilative effect were more than visual or auditory perception processing, which can be best accounted by numerical priming. Since the rating of the 2-back trial showed an assimilative effect on the current rating, assimilation could not be due to a motor effect of immediate response repetition. Earlier studies had confirmed the use of anchoring-and-adjustment heuristic when people make estimation of something they were unfamiliar with (e.g., [22,57]). In this study, when judging the current stimulus, participants referred to the previous judgment and regarded it as an anchor. The assimilation was the result of the insufficient adjustments of the anchor. Finally, we conclude that the contrastive effect depends on visual processing or auditory processing, because no contrastive effect was found between adjacent trials of different sensory modalities, but did occur for the 2-back trial of the same sensory modality.

Some may argue that we should not neglect the 2-back trials in the former 4 experiments. Taking 2-back trials in Experiment 1 & 2 & 4 into account, we found significant assimilative effect but no contrastive effect of 2-back trials, with sequential effects of 1-back trials little affected. Besides, the assimilative effect of 1-back trial is significantly stronger than that of 2-back trial, which downplays the assimilation of 2-back. For instance, estimated beta of R_t-1_ and R_t-2_ were 0.18 (*SE* = .02; *t* = 8.04, *p*< .001) and 0.07 (*SE* = .03; *t* = 2.91, *p* = .006) respectively (*t* = 7.82, *p*< .001) in Experiment 1. But we assume that it is hard to explain the assimilative effect of 2-back trial because ratings of 1-back trial and 2-back trial correlate highly, which causes multicollinearity in the regression modelling. Second, the regression modelling is redundant when we take 2-back trials into account. Third, what we are interested in is not the sequential biases of 2-back trials in Experiment 1–4. Therefore, we do not think it necessary to take 2-back trials into consideration when we examine sequential effect.

## General discussion

Sequential biases are prevalent in sequential judgment tasks in the domain of psychophysics. However, in the domain of subjective decision making such as facial attractiveness judgments, there is also no consistent conclusion about the sequential biases *per se* as well as how the sequential biases occur. In this paper, we conducted five experiments to figure out how we made a decision in a sequential subjective judgment task and how the sequential biases came into being.

In the Experiments 1 and 2, we examined the existence of sequential biases in face attractiveness and ringtone agreeableness judgment tasks and results revealed that both assimilative and contrastive effects were significant. Taking the face attractiveness judgment task for example, if we judged the previous face as relatively attractive or if the previous face was relatively unattractive, we tended to rate the current face assimilating to the previous rating (assimilation) but away from the value of the previous stimulus (contrast).

In Experiments 3–5, we examined the influence of feedback provision, participant’s response modes, and cross-modal stimuli on sequential effects. All of these variables exerted significant impact on the sequential biases.

### Bias from the previous stimuli

As for the aftereffect of the previous stimulus, our results showed that the value of the previous stimulus negatively predicted the rating of the current stimulus. The finding of contrastive effect is in line with previous studies. As for facial attractiveness judgment, if the previous is relatively attractive, then we tend to give a lower evaluation than in the normal situation, and vice versa. In Experiment 1, when all other variables were held constant, participants rated the current face 0.23 rating units less attractive if the previous stimulus value was one rating unit more attractive than the sample mean. In Experiment 2, we also found that if the preceding ringtone was less agreeable than the sample mean, participants rated the agreeableness of current ringtone more agreeable than on usual condition, which matches the pattern found in face attractiveness judgment. What surprised us was that the contrastive effect was robust either in the judgment of face attractiveness or ringtone agreeableness. We believed that bias from the previous stimuli would also occur when people make decisions of the sequential items in the domain of subjective judgment.

Nonetheless, contrastive effects never occurred when the continuous items were of different sensory modalities. In Experiment 5, when participants were required to judge the face attractiveness and ringtone agreeableness alternatively, no significant contrastive effect was found from the previous 1-back stimulus. That is, when the continuous stimuli were not of the same modality (even the same category), the contrastive effect disappeared. Conversely, the value of 2-back stimulus negatively predicted the rating of agreeableness on the current ringtone. As for face attractiveness judgment, the same 2-back contrastive effect was found at marginal significant level. The above findings convinced us that the absence of the contrastive effect was a result of the change of stimulus modality or category. For example, contrastive effect on the judgment of facial attractiveness depends on the visual perception processing, which is in agreement with the perceptual interpretation of face aftereffects [34,47].

In Experiment 3, we clarified that the contrastive effect was almost eliminated with the true value provided as feedback after participants made their judgments. The influences of feedback on the sequential effects were widely studied in psychophysical experiments [2,5,51]. Researchers also demonstrated that the provision of feedback produced a masked effect of perception of the current stimuli [27]. In our study, contrastive effect resulting from the previous stimulus was not found with the provision of feedback, which suggested that the perceptual processing of the previous face was essential to sequential bias. And the finding was consistent with what was found in psychophysical experiments (e.g., [2])as well as in more real-world setting research (e.g., [11,30]).

More specifically, we assumed that the contrastive effect occurred unconsciously. Pegors et al. (2015) explored the sequential effects with a novel sequential rating paradigm in which participants alternated their judgments between face attractiveness and hair darkness. According to their results, the contrastive effect was enhanced as the display duration of the previous face increased, suggesting the visual perception explanation instead of cognitive remapping (remap certain face types to the rating scale numbers). Although it was the dimension of hair darkness that participants judged in the previous trial, the dimension with minor attention, face attractiveness, still had a negative prediction on the attractiveness of the current face, which implied the subconscious process of face perception. In Experiment 5, the attractiveness value of 2-back trial negatively (although marginally significant) predicted the current attractiveness rating, which also implied that unconscious visual perception of the previous face was important to contrastive effect. We also found the contrastive effect of the 2-back on ringtone agreeableness rating. Apparently, the 2-back contrastive found on the ringtone agreeableness rating was much stronger than that on the face attractiveness rating. Why the aftereffect of auditory stimulus is stronger than that of visual stimulus needs future exploration.

Recent research has provided evidence that facial attractiveness can be processed in the complete absence of consciousness [36], on account of the fact that attractive faces enjoyed the privilege of breaking suppression and reaching consciousness earlier. Therefore, it’s not hard to understand why contrastive effect and assimilative effect exist simultaneously because they have totally different mechanisms.

In conclusion, when we made decision of the items in a series, we were likely to judge the current stimulus in a non-optimal way resulting from the previous item *per se*, which exerted a negative effect.

### Bias from the previous responses

In Experiments 1 and 2, both contrastive effect and assimilative effect were found simultaneously. (Although the ratings of the preceding stimuli and the values of them were positively correlative, their effects on the current ratings were in opposite directions). Taking the judgment of face attractiveness for example, the current facial attractiveness rating assimilates toward the previous rating in our study, which is parallel to the earlier studies [32,37,56]. Specifically, participant rated the current face 0.21 rating units more attractive than it should have been if the previous face was rated one unit more attractive above average. However, the assimilative effect diminished significantly in the presence of trial-by-trial feedback, which was also similar to the previously studies (e.g., [11,30]). It was well-known that participants used the previous judgment as a point of reference on the current trial in psychophysical research. Our findings just corroborated that feedback gave a masked effect of the previous trial since the strength of assimilative effect was reduced with feedback provision, suggesting that sequential effect may share the mechanism as the sequential effects found in the psychophysical tasks.

One of the interesting findings is that the way participants made their judgments influenced the strength of assimilative effect. Compared to traditional key-pressing response, when participants made judgments of the face attractiveness orally, weaker assimilative effects resulting from the previous response were found. It was obvious that participants were able to make their ratings freely with verbal protocol which was recommended by some researchers such as Ericsson and Simon [52]. Verbal protocol can largely avoid the tendency to repeat the previous response. Thus, the trend of action repetition may contribute partially to the assimilative effect.

Taubert et al. (2016) suggested assimilation to the previous trial results from perceptual processing [55] as the assimilative effect was diminished when the two consecutive faces were not at the same orientation, which disrupted almost all perceptual processes underlying face perception. However, we found a significant cross-modality assimilative effect of previous response when the two consecutive stimuli were not from the same modality in Experiment 5. This cross-modality assimilation was not the result of repetition because oral response was required in Experiment 5, which suggested that the number priming contributed more to the assimilation. In addition, repetition cannot explain why the rating from the 2-back trial positively predicted the current rating. And finally, a reduced assimilative effect was found in Experiment 3 when we provided the trial-by-trial feedback that destroyed the priming of the previous rating. Thus, the insufficient adjustment of the anchor (the previous rating) may lead to the assimilative effect. Taking all factors into account, we argue that the mechanisms of assimilative effect is anchoring effect of the previous judgment on the same kind of stimulus, which are independent of perceptual processing. And assimilative effect can be strengthened by response repetition and numerical priming. Therefore, beyond the bias from the previous stimulus, our judgment of the previous stimulus will also mislead us to the non-optimal choice in the sequential subjective judgment.

As to the overall sequential effect of the previous stimulus and previous response on current rating, we take the face attractiveness judgment experiment as an example. On the one hand, the current face will be given lower rating, if the face antecedent to it is relatively high in attractiveness. This is how the contrastive effect works, and it is the stimulus *per se* that acts as a reference for comparison. On the other hand, due to the high rating given on the previous trial, we will be less likely to give an extremely low score that brings forth a huge contrast to the current face. This is how the assimilative effect works, and it is our previous response that anchors the judgment.

On this article, we contributed to the existing literature on sequential effect in the following ways: First, we broadened the domain of the sequential effects by originally drawing attention to auditory stimuli, extending the concept of sequential effects to subjective decision making, as well as examining its cross-modal ability. Second, we solved the contradiction in previous research with empirical evidences. Our results provided support for the visual (or auditory) perception theory in contrastive effect, as well as the influence of anchoring effect (of the same kind of stimulus) in assimilative effect, which can be strengthened by response repetition and numerical priming. Overall, the study helps us understand how we make a sub-optimal decision on the sequential judgment.

There are some limitations of the study. First, the average of subjective ratings is only the central tendency of subjective values. Future study can try to introduce the objective value of face attractiveness by using computational models of face attractiveness. Second, the scope of our attention on sequential biases is currently limited to behavioral indicators. Further research is warranted to cast light on the issues such as mechanisms of sequential effects from a more in-depth neuroscience perspective, and the application prospect of the effects observed in the laboratory to real-world settings. Besides, future research can try to explore under what conditions one of the effects plays a predominant role over the other.

## Conclusion

In the current study, we first demonstrated that our subjective judgment on the current stimulus was biased by both previous stimuli and previous judgments. That is, contrastive and assimilative effects occurred simultaneously on the sequential subjective judgments, and the pattern of the observed sequential biases fit the regression model derived from psychophysical research. We also proved that contrastive effect resulted from low-level perceptual processing of the previous stimuli while the essence of assimilative effect was anchoring of the previous judgments of the same kind of stimulus, which can be strengthened by low-level response repetition as well as numerical priming of the previous rating.

## Supporting information

S1 Data Data underlying this study. (ZIP)
