# Supplementary material for: Sequential biases on subjective judgments: Evidence from face attractiveness and ringtone agreeableness judgment
Source: PLoS One. 2018 Jun 11;13(6):e0198723. doi: 10.1371/journal.pone.0198723 (PMC5995378; doi:10.1371/journal.pone.0198723)
Supplement: S1 Data — (ZIP) [file pone.0198723.s001.zip › all the data/ATTENTION!.docx]

For all the Excel files, if there are only two columns of data, then Column 1 represents Rt, and Column 2 represents St. Else, Column 1 represents Rt, Column 3 represents St.
